# Manufacturing of Ni-Co-Fe-Cr-Al-Ti High-Entropy Alloy Using Directed Energy Deposition and Evaluation of Its Microstructure, Tensile Strength, and Microhardness

**DOI:** 10.3390/ma17174297

**Published:** 2024-08-30

**Authors:** Ho-In Jeong, Jae-Hyun Kim, Choon-Man Lee

**Affiliations:** Mechatronics Research Center, Changwon National University, Changwon 51140, Republic of Korea; jhi8443@changwon.ac.kr

**Keywords:** additive manufacturing, direct energy deposition, high-entropy alloy, phase analysis, material properties

## Abstract

High-entropy alloys (HEAs) have drawn significant attention due to their unique design and superior mechanical properties. Comprising 5–35 at% of five or more elements with similar atomic radii, HEAs exhibit high configurational entropy, resulting in single-phase solid solutions rather than intermetallic compounds. Additive manufacturing (AM), particularly direct energy deposition (DED), is effective for producing HEAs due to its rapid cooling rates, which ensure uniform microstructures and minimize defects. These alloys typically form face-centered cubic (FCC) or body-centered cubic (BCC) structures, contributing to their exceptional strength, hardness, and mechanical performance across various temperatures. However, FCC-structured HEAs often have low yield strengths, posing a challenge for structural applications. In this study, a Ni-Co-Fe-Cr-Al-Ti HEA was manufactured using the DED method. This study proposes that the addition of aluminum and titanium creates a γ + γ′ phase structure within a multicomponent FCC-HEA matrix, enhancing the thermal stability and coarsening the resistance and strength. The γ′ phase with an ordered FCC structure significantly improves the mechanical properties. Analysis confirmed the presence of the γ + γ′ structure and demonstrated the alloy’s high tensile strength and microhardness. This approach underscores the potential of AM techniques in advancing HEA production for high-performance applications.

## 1. Introduction

High-entropy alloys (HEAs) have gained considerable attention due to their innovative design and outstanding mechanical properties [1]. HEAs consist of 5~35 at% of five or more elements with similar atomic radii, resulting in high configurational entropy, significant lattice distortion, and reduced diffusion rates [2]. HEAs are typically characterized by configurational entropies exceeding 1.5R (where R is the gas constant) [3]. The configurational entropy equation is shown in Equation (1).

(1) $$\Delta S_{mix}=-R\sum_{i}^{N}x_{i}\ln x_{i}$$

where $\Delta s_{mix}$ is the constituent entropy, N is the number of elements, and $x_{i}$ is the entropy of each element. Due to their high entropy, HEAs tend to form single-phase solid solutions rather than intermetallic compounds. This unique composition and microstructure give HEAs exceptional mechanical properties. HEAs generally exhibit a single-phase face-centered cubic (FCC) or body-centered cubic (BCC) structure [4]. Unlike typical alloys, HEAs form a concentrated solid solution. Consequently, HEAs exhibit high strength and hardness at room temperature, along with excellent mechanical performance at both low and high temperatures [5].

In particular, the extraordinary ductility and fracture toughness of FCC-structured HEAs at ambient temperature and cryogenic temperatures have garnered significant attention [6,7,8,9,10]. The low yield strength of FCC-structured HEAs, which is typically just 200 MPa in the as-cast form, is a significant disadvantage, as it is significantly less than the necessary strength of metallic structural materials [11,12]. To overcome these limitations in mechanical properties, a proposed method is to manufacture an HEA composed of a multicomponent FCC-HEA matrix with an ordered FCC structure (L1_2_) precipitated phase, i.e., the γ′ phase, by adding Al + Ti. An HEA based on a γ + γ′ alloy system exhibited excellent thermal stability, coarsening resistance, and strength retention around a temperature of 800 °C [10,11,12,13]. The γ′ phase is an ordered phase with an almost exact stoichiometric composition ((Ni, Co)3(Ti, Al)) in the Ni-Co-Fe-Cr-Al-Ti HEA, with γ′ depending on the Al + Ti content [14]. Numerous studies have been conducted on the γ′ phase’s formation and mechanical properties [15,16]. A high content of Al + Ti leads to the formation of precipitate phases such as Ni_2_AlTi. When the Al/Ti ratio is high, the NiAl precipitates, whereas a low Al/Ti ratio results in the precipitation of Ni_3_Ti [17]. Therefore, in Ni-Co-Fe-Cr-Al-Ti alloys, the composition should be adjusted to achieve a γ + γ′ phase structure. It is crucial to control the chemical composition to ensure that only the desired secondary phase forms without any other unwanted phases.

Most research on HEAs has focused on evaluating their fundamental properties by creating ingots through arc melting and casting [18]. HEAs require a rapid cooling rate to achieve single-phase solid solutions [19,20]. As the size of the HEA increases, differences in the internal and external cooling rates make it challenging to maintain uniform microstructures, thus limiting the scalability of HEAs [21]. Additive manufacturing (AM), which completes processes in a relatively short time, is well suited for producing HEAs [22,23,24,25]. Direct energy deposition (DED) is commonly employed in AM [26]. The fast solidification rate of DED is beneficial for grain refinement and can prevent component segregation [27]. Additionally, the efficient heat source in DED results in a narrow heat-affected zone (HAZ) and a low dilution rate of the base material. These characteristics primarily reduce cracks, deformations, and metallurgical changes in the base metal, leading to fewer defects and improved production quality compared to other methods [28]. Therefore, through the application of the DED process in the AM of HEAs, HEAs with excellent performance can be produced with a very high production efficiency [29,30].

**Figure 1:**
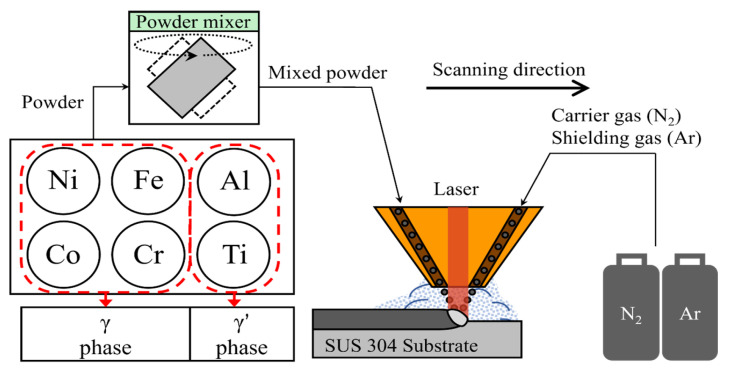
The schematic for the DED of Ni-Co-Fe-Cr-Al-Ti HEA.

In this study, a Ni-Co-Fe-Cr-Al-Ti HEA with an optimally designed composition was deposited using DED. Figure 1 shows a schematic diagram of the DED of the Ni-Co-Fe-Cr-Al-Ti HEA. Before deposition, the phase diagram of the Ni-Co-Fe-Cr-Al-Ti HEA was analyzed. For the DED of the Ni-Co-Fe-Cr-Al-Ti HEA, powders of each element were mixed to prepare a mixed Ni-Co-Fe-Cr-Al-Ti powder, which was deposited on SUS 304 substrates. Scanning electron microscopy (SEM), X-ray diffraction (XRD), and electron backscatter diffraction (EBSD) analyses were conducted to verify the microstructure of the deposited Ni-Co-Fe-Cr-Al-Ti HEA. The microstructure of the Ni-Co-Fe-Cr-Al-Ti HEA exhibited a γ + γ′ structure, and it was verified that a γ′ phase composed of Al + Ti was added. In addition, the tensile strength and microhardness of the deposited Ni-Co-Fe-Cr-Al-Ti HEA were measured. The Ni-Co-Fe-Cr-Al-Ti HEA constructed of the γ′ phase exhibited good mechanical properties.

## 2. Phase Analysis

**Figure 2:**
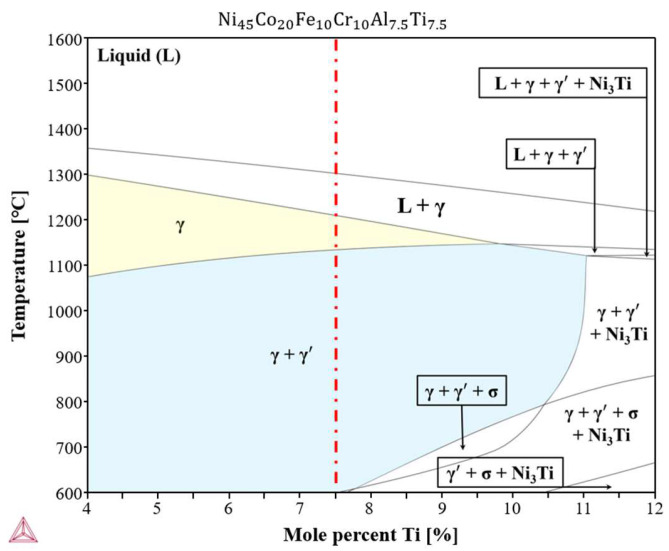
The phase diagram of Ni-Co-Fe-Cr-Al-Ti high-entropy alloy.

Figure 2 shows a phase diagram of the Ni-Co-Fe-Cr-Al-Ti high-entropy alloy. The phase diagram of the Ni-Co-Fe-Cr-Al-Ti high-entropy alloy was developed and analyzed using the Thermo-Calc software (version 2022a) and the TCFE12 database under equilibrium conditions. The Ni-Co-Fe-Cr-Al-Ti high-entropy alloy was composed of 45.0 at% Ni, 20.0 at% Co, 10.0 at% Fe, 10.0 at% Cr, 7.5 at% Al, and 7.5 at% Ti, respectively. The composition of the Ti varied from 4% to 12% along the *x*-axis, and the temperature varied from 600 K to 1600 K along the *y*-axis. The Ni-Co-Fe-Cr-Al-Ti high-entropy alloy was completely melted at temperatures above 1300 °C, and the γ phase with the FCC structure appeared as the temperature decreased. The γ′ phase of the FCC structure, which was predicted to occur upon the addition of Ti-Al, appeared at 1113 °C. As a result of the phase analysis, it was found that the Ni-Co-Fe-Cr-Al-Ti high entropy alloy, which was designed with the optimal values, did not appear in precipitation phases other than the γ + γ′ phase. Table 1 shows the chemical composition of the Ni-Co-Fe-Cr-Al-Ti high-entropy alloy.

## 3. Experimental Procedure

### 3.1. Experimental Setup and Materials

**Figure 3:**
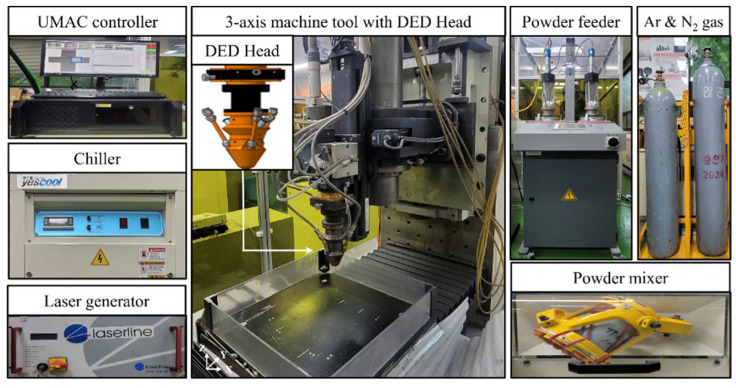
The experimental setup for DED.

Figure 3 shows the experimental setup for the DED. Using a machine tool and a DED head, the DED equipment for the deposition of the Ni-Co-Fe-Cr-Al-Ti was manufactured. The machine tool’s X, Y, and Z axes had strokes of 500, 500, and 300 mm, respectively. A diode laser (LDM 2000, Laserline, GmbH, M Mulheim-Karlich, Germany) with a power output of 2 kW, a beam diameter of 3 mm, a focal length of 198 mm, and a wavelength of 980 nm was coupled to the DED head mounted on the machine tool [31]. Additionally, the DED equipment included a 780 W water chiller (YRC-1A, Yescool, Bucheon, Republic of Korea) for laser cooling [32]. A 1000 rpm rotating powder mixer (MI-2T, DIM-NET, Incheon, Republic of Korea) was used to create the Ni-Co-Fe-Cr-Al-Ti powder [19,20]. Each Ni, Co, Fe, Cr, Al and Ti powder used in this study was a pure powder supplied by MK Metal Corporation (Pyeongtaek, Republic of Korea). The powder was delivered to the DED head using a dual-head powder feeder (Twin 150, Oerlikon Metco, Pfaeffikon, Switzerland). A Universal Machine and Automation Controller (UMAC) oversaw the DED system controls, and air-infiltration-induced oxidation was prevented throughout the DED system by sealing it off. The carrier and shielding gases for the powder delivery were nitrogen and argon, respectively. The nitrogen and argon gases were purchased from HANSCO, a Korean company (Daejeon, Republic of Korea), at a 99.7% purity. Nitrogen gas was used as the process gas because of its several advantages. It is an inert gas that prevents the oxidation of metal powders during the deposition process. Since titanium and aluminum are prone to oxidation, the use of nitrogen therefore mitigated the oxidation process. By minimizing the oxidation process, the quality of the deposited material was improved, leading to better mechanical properties. In addition, it also dissipated the heat generated during the DED process. Moreover, it allowed for a smooth feed of the powder into the melt pool, resulting in a more uniform deposition [33].

In our DED process for fabricating the HEA, the interaction between nitrogen and the alloy components was carefully considered. The selected HEA system was thermodynamic stable, which reduced the possibility of it reacting with nitrogen and thus forming nitrides or other nitrogen-containing compounds [34]. In addition, we selected the processing temperature and time during the DED process to prevent nitrogen diffusion and reaction. In the upcoming sections, our XRD and SEM results also confirm the absence of secondary phases. These factors collectively demonstrate that the nitrogen primarily served as a process gas without reacting with the alloy elements to form additional compounds [35,36].

**Figure 4:**
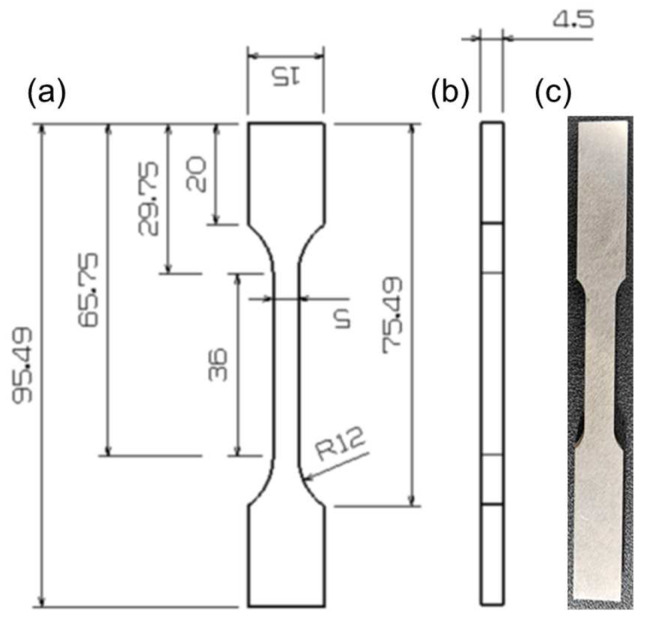
The tensile test specimens as per ASTM E8/E8M-18 standard: (**a**) front view, (**b**) side view, and (**c**) fabricated sample. Unit: mm.

The X-ray diffraction (XRD) analysis of the developed HEA was conducted through XRD (SmartLab SE, Rigaku Corporation, Tokyo, Japan) using Cu radiation (λ = 1.54 Å, 40 kV, 50 mA) to confirm the phase structure. Electron backscatter diffraction (EBSD) analysis was performed using an EBSD detector (Clarity, EDAX, Mahwah, NJ, USA) to investigate the grain size. The morphology and energy-dispersive X-ray spectroscopy (EDS) analysis was performed using a silicon drift detector (SDD; Octane, EDAX). In addition, the tensile test was performed using a tensile tester (M5582, INSTRON, Norwood, MA, USA). The specimens were manufactured to a thickness of 4.5 mm as per the ASTM E8/E8M-18 standard [37], as shown in Figure 4. The specimen for tensile testing was fabricated using Wire Electrical Discharge Machining (Wire EDM) followed by polishing the surface with a fine grinder for better results. The microhardness analysis was carried out using a Mitutoyo HM-200 Vickers hardness tester (Aurora, IL, USA). Hardness measurements were performed according to the deposited height from the SUS 304 substrate, with an average of five readings per height recorded.

### 3.2. DED Method and Conditions

To decide the DED temperature, a phase analysis was conducted on the Ni-Co-Fe-Cr-Al-Ti HEA. Above the melting point of 1300 °C, Ni-Co-Fe-Cr-Al-Ti HEA transformed from a liquid state to a solid state in the γ phase with decreasing temperature. During solidification, the γ′ phase grew at 1113 °C. Since the designed Ni-Co-Fe-Cr-Al-Ti HEA formed a single-phase solid solution with an FCC structure, the DED temperature was selected at 1300 °C because only complete dissolution was considered without considering the formation of a secondary phase.

**Figure 5:**
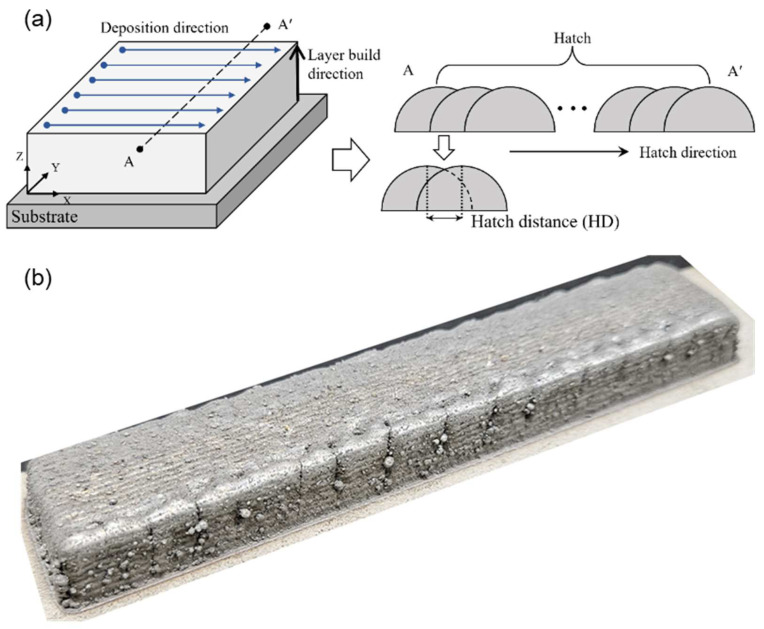
(**a**) Schematic of the DED strategy showing the deposition direction and hatch spacing; (**b**) the fabricated HEA.

The Ni-Co-Fe-Cr-Al-Ti HEA was manufactured by mixing each of the powders, which had a diameter of 45 and 150 μm and a 99.9% purity, in a powder mixer for four hours at the precise ratio indicated in the phase diagram and then depositing the mixture onto the SUS 304 substrate. The laser power, scanning speed, powder feed rate, hatch distance, and shield gas flow rate were among the DED processing conditions. The hatch distance was determined with the DED’s quality in mind. The mechanical characteristics can be diminished by interior porosity and cracks caused by an excessive hatch spacing. On the other hand, a smaller hatch distance results in an enlarged melt pool and an uneven layer, which raises internal stresses and lowers the production efficiency. Figure 5a shows a schematic of the DED strategy. The HEA was deposited along the positive *x*-axis with a laser velocity of 10 mm/s, a powder feed rate of 7 g/min, and a hatch spacing of 1 mm. As the deposition continued, the layer built up along the *z*-axis, with a total of 11 layers, thus fabricating the desired specimen, as shown in Figure 5b. The hath distance was selected to reduce the residual stress, control the grain size, and enhance the bonding between layers. Table 2 lists the processing conditions of the DED.

## 4. Results and Discussion

### 4.1. XRD Analysis

**Figure 6:**
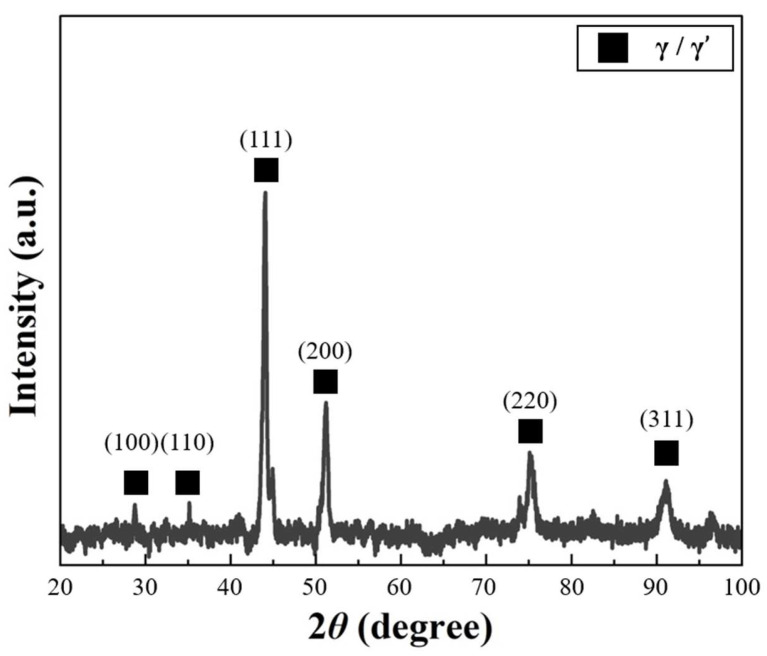
The results of the XRD analysis.

The XRD data were collected in the 2-theta range of 20°–100° in 0.02° step increments at 20 °C. Figure 6 shows the results of the XRD analysis. The XRD pattern exhibited peaks corresponding to the FCC structure. The detected (100) superlattice peak corresponded to the ordered FCC (L12) structure, confirming the presence of the γ′ phase [15]. Therefore, through the FCC peak and superlattice peak detected in the XRD pattern, the Ni-Co-Fe-Cr-Al-Ti HEA was expected, in the FCC single-phase structure, to represent the γ + γ′ phase.

### 4.2. SEM and EDS Analysis

**Figure 7:**
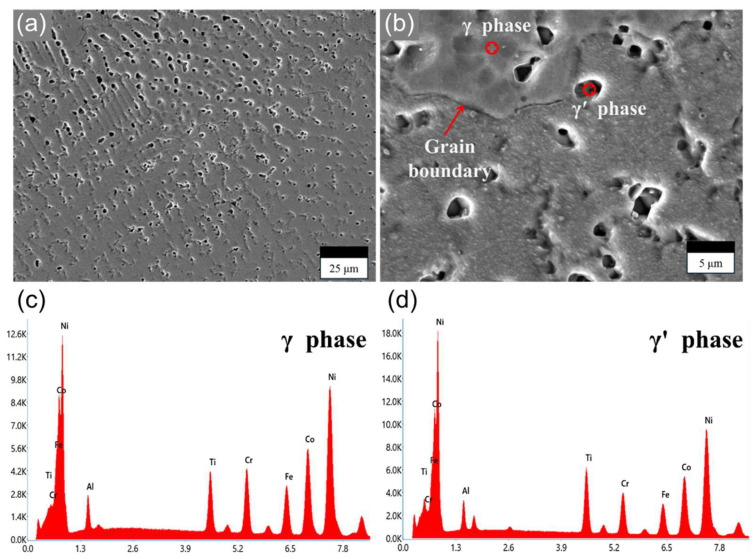
The SEM images of Ni-Co-Fe-Cr-Al-Ti HEA: (**a**) ×1000 magnification, (**b**) ×5000 magnification. EDS analysis confirming the elemental composition in (**c**) γ and (**d**) γ′ phases.

Figure 7a,b show the SEM images of the Ni-Co-Fe-Cr-Al-Ti HEA. The matrix phase of the Ni-Co-Fe-Cr-Al-Ti HEA appeared as the γ phase of the FCC structure, and the γ′ phase of the ordered FCC structure was confirmed to be widely distributed in the form of dot-like pores. In addition, no additional precipitated phases appeared on the SEM images, and it was confirmed that the grain size of the γ phase was coarse. For the component analysis of the Ni-Co-Fe-Cr-Al-Ti HEA and each phase, a component analysis of the entire area and each phase was conducted.

Figure 7c,d show the results of the EDS analysis of the deposited Ni-Co-Fe-Cr-Al-Ti HEA for the γ and γ′ phases, respectively. The analysis confirmed that the Ni-rich γ phase solid solution with Al and Ti was formed first, followed by the formation of the γ′ phase solid solution with a (Ni, Co)3(Ti, Al) composition. The composition of the Ni-Co-Fe-Cr-Al-Ti HEA was like that of the designed composition; however, there was a compositional difference of 1~2% for each element. Table 3 shows the chemical composition of the developed HEA for both the γ and γ′ phases.

**Figure 8:**
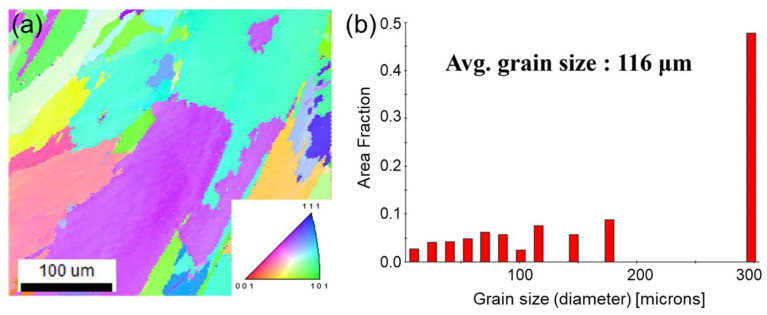
(**a**) The results of the electron backscatter diffraction analysis inverse pole figure map (IPF) and (**b**) the grain size plot of Ni-Co-Fe-Cr-Al-Ti HEA.

The Ni-Co-Fe-Cr-Al-Ti HEA exhibited a single solid solution with an FCC structure with an average particle size of 116 μm. Figure 8a shows the electron backscatter diffraction analysis inverse pole figure map (IPF), and Figure 8b shows the grain size of the Ni-Co-Fe-Cr-Al-Ti HEA. The developed HEA exhibited a significantly large grain size, but no other precipitated phases were found. This is thought to have been caused by the γ phase becoming coarser because of the high-temperature deposition. The recommended deposition temperature for the Ni-Co-Fe-Cr-Al-Ti HEA was determined to be 800 °C, which indicates a high γ′ phase fraction and a low coarsening rate [14]. It is predicted that if the size of the phase was reduced, it would exhibit slightly better mechanical properties.

The major reasons for the uneven grain size distribution could be the effect of variations in the processing parameters, such as the processing temperature, cooling rate, and environmental conditions. In addition, the heterogeneous nucleation and growth rate of the different elements could have also led to an uneven grain size throughout the material. Moreover, the variations in the composition also affected the nucleation sites and growth rates of the different phases, leading to varied grain sizes. HEAs can undergo phase transformations during processing. These transformations can alter the grain size distribution by changing the nucleation and growth rates of the alloy phases.

### 4.3. Tensile Strength

**Figure 9:**
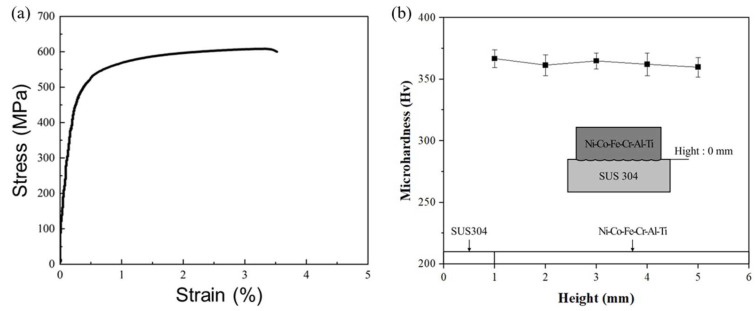
(**a**) The tensile test results of Ni-Co-Fe-Cr-Al-Ti HEA, (**b**) the microhardness distributions of Ti-Nb-Cr-V-Ni deposited on the substrates.

The yield strength and ultimate tensile strength of the Ni-Co-Fe-Cr-Al-Ti HEA were 514 MPa and 605 MPa, respectively. Figure 9a shows the tensile strength test results of the Ni-Co-Fe-Cr-Al-Ti HEA. The tensile strength of the Ni-Co-Fe-Cr-Al-Ti HEA was lower than that of Inconel 718 due to the coarsening of the microstructure [38]. However, it is anticipated that the mechanical properties of the Ni-Co-Fe-Cr-Al-Ti HEA could be enhanced with post-processing treatments like heat treatment [39].

### 4.4. Microhardness

Figure 9b shows the microhardness distribution for the deposited Ni-Co-Fe-Cr-Al-Ti HEA. The developed HEA exhibited a consistent hardness of approximately 370 HV, showing no variation with height. This value exceeds the typical hardness of wrought Inconel 718, which is around 350 HV [40]. The Ni-Co-Fe-Cr-Al-Ti HEA’s grain size appeared to be relatively coarse but was distributed higher than that of general alloys. It is anticipated that after heat treatment to refine the size of the phase, the HEA would exhibit better mechanical properties [41].

## 5. Conclusions

In this study, a Ni-Co-Fe-Cr-Al-Ti HEA was deposited using DED. The phase diagram of the Ni-Co-Fe-Cr-Al-Ti HEA was analyzed in terms of the deposition of Ni-Co-Fe-Cr-Al-Ti. For the DED of the Ni-Co-Fe-Cr-Al-Ti HEA, powders of each element were mixed to prepare a mixed Ni-Co-Fe-Cr-Al-Ti powder, and this was deposited on SUS 304 substrates. The powders of each element of the Ni-Co-Fe-Cr-Al-Ti HEA were mixed and deposited on substrates. The microstructure, tensile strength, and hardness of the deposited Ni-Co-Fe-Cr-Al-Ti alloy were measured. The primary findings of this study are as follows:

1. As a result of the phase analysis of the Ni-Co-Fe-Cr-Al-Ti HEA, the γ phase of the FCC structure appeared as a matrix phase in the Ni-Co-Fe-Cr-Al-Ti HEA, and the γ′ phase of the ordered FCC structure additionally appeared. In addition, the Ni-Co-Fe-Cr-Al-Ti HEA, designed with optimal values, was found to not appear in the precipitated phases other than the γ + γ′ phase.
2. The matrix phase of the Ni-Co-Fe-Cr-Al-Ti HEA appeared as the γ phase of the FCC structure, and the γ′ phase of the aligned FCC structure was confirmed to be widely dispersed in the form of dots similar to pores. Furthermore, the SEM pictures showed no more precipitated phases, and the coarse grain size of the γ phase was verified.
3. The tensile test of the Ni-Co-Fe-Cr-Al-Ti HEA exhibited a yield strength of 514 MPa and an ultimate tensile strength of 631 MPa, using ASTM E8/E8M-18 standard specimens. The Ni-Co-Fe-Cr-Al-Ti HEA’s tensile strength was lower than Inconel 718 due to microstructural coarsening. However, post-processing treatments like heat treatment are expected to improve the mechanical properties.
4. The Ni-Co-Fe-Cr-Al-Ti HEA deposited via DED exhibited a consistent microhardness of approximately 370 HV, and no hardness change with height was observed. This surpassed the 350 HV of machined Inconel 718. Despite its coarse particle size, it exhibited high hardness, and its mechanical properties are expected to be improved through post-treatment heat treatment.

With precise phase analysis and post-treatment procedures added, this work on the production of an HEA with a Ni-Co-Fe-Cr-Al-Ti composition utilizing DED can be utilized as a novel HEA manufacturing method.

## Figures and Tables

**Table 1:** The chemical composition of Ni-Co-Fe-Cr-Al-Ti high-entropy alloy.

Element (at%)	Ni	Co	Fe	Cr	Al	Ti
	45.0	20.0	10.0	10.0	7.5	7.5

**Table 2:** The processing conditions of DED.

Process Parameter	Value
Laser power (W)	1000
Laser velocity (mm/s)	10
Powder feed rate (g/min)	7
Hatch spacing (mm)	1.0
Argon gas flow (L/min)	20
Nitrogen gas flow (L/min)	5

**Table 3:** The chemical compositions of structure constituents for the Ni-Co-Fe-Cr-Al-Ti HEA.

Element, at%	Ni	Co	Fe	Cr	Al	Ti
Designed	45.0	20.0	10.0	10.0	7.5	7.5
γ phase	49.3	24.3	9.4	8.8	4.5	3.7
γ′ phase	44.8	20.4	8.2	8.5	6.8	11.3
Area	46.3	20.5	9.8	9.1	7.1	7.2

## Data Availability

The raw data supporting the conclusions of this article will be made available by the authors on request.
